# Rescue Cervical Cerclage in a Twin Pregnancy With Short Cervical Length or Cervical Dilatation: A Case Report

**DOI:** 10.7759/cureus.113224

**Published:** 2026-07-23

**Authors:** Fariha Altaf, Biza Akbar

**Affiliations:** 1 Obstetrics and Gynaecology, Tameside and Glossop Integrated Care NHS Trust, Manchester, GBR; 2 Urogynaecology, Tameside and Glossop Integrated Care NHS Trust, Manchester, GBR

**Keywords:** cervical cerclage, cervical insufficiency, pprom, preterm birth, short cervical length, twin pregnancy, uterine fibroids

## Abstract

Preterm birth is a leading cause of neonatal morbidity and mortality worldwide, with the risk being substantially higher in multiple pregnancies. Twin gestations are associated with several maternal and foetal risk factors that contribute to spontaneous preterm birth. Although cervical cerclage is an established intervention for cervical insufficiency in singleton pregnancies, its role in preventing preterm birth in twin pregnancies remains controversial, and its use continues to be the subject of ongoing research. This case report details the management of a woman with a twin pregnancy who presented with abdominal pain. She had one anterior wall and one fundal fibroid; however, they did not cause any compression effects on the endometrial cavity. Rescue cerclage was performed at 17 weeks and three days of gestation following thorough counseling on potential risks and benefits. The patient chose to have the cerclage, received continuous multidisciplinary antenatal care, and ultimately delivered at 27 weeks and three days via emergency caesarean section due to premature rupture of membranes. Throughout her care, informed discussions supported shared decision-making.

## Introduction

Preterm birth (PTB) is a serious global health issue, accounting for a large proportion of neonatal mortality and long-term health problems. The risk is even greater in twin pregnancies, where preterm birth rates are significantly higher than in singletons [1,2]. Various interventions have been investigated to prevent preterm birth, such as administration of progesterone, placement of cervical cerclage, and an Arabin pessary [3]. Nevertheless, the efficacy of these measures in multiple gestations is still unclear.

Twin pregnancies are classified by chorionicity and amnionicity, with dichorionic diamniotic (DCDA) twins being the most common type resulting from the fertilisation of two separate eggs (dizygotic twins). DCDA twins have separate placentas and amniotic sacs, which generally lowers some risks compared to monochorionic twins, though the risk of preterm birth remains high. Cervical incompetence, also known as cervical insufficiency, is a condition in which the cervix painlessly dilates and shortens during the second trimester, increasing the risk of mid-trimester pregnancy loss or preterm delivery [1]. It may be diagnosed based on clinical history or ultrasound findings of painless cervical dilation or shortening, especially in women with a history of recurrent pregnancy loss or preterm birth.

Cervical cerclage is a surgical procedure performed to reinforce the cervix with a suture, typically between 12 and 24 weeks of gestation [2]. The most common technique is the McDonald procedure, which involves placing a purse-string suture around the cervix via the vagina. Cerclage may be indicated in women with a history of cervical insufficiency, findings of cervical shortening, or painless dilation on examination. Notably, the role of cerclage in twins is controversial, with current research indicating that its benefit may be confined to certain high-risk cases, particularly those with evidence of cervical shortening or early dilation [4,5].

This case highlights the use of emergency McDonald cerclage in a DCDA twin pregnancy complicated by advanced cervical shortening and early cervical dilatation, contributing to the growing body of evidence supporting carefully selected use of cerclage in high-risk twin gestations.

The abstract was previously presented as an e-poster at the RCOG World Congress, held in London from 23-25 June, 2025.

## Case presentation

**Figure 1:**
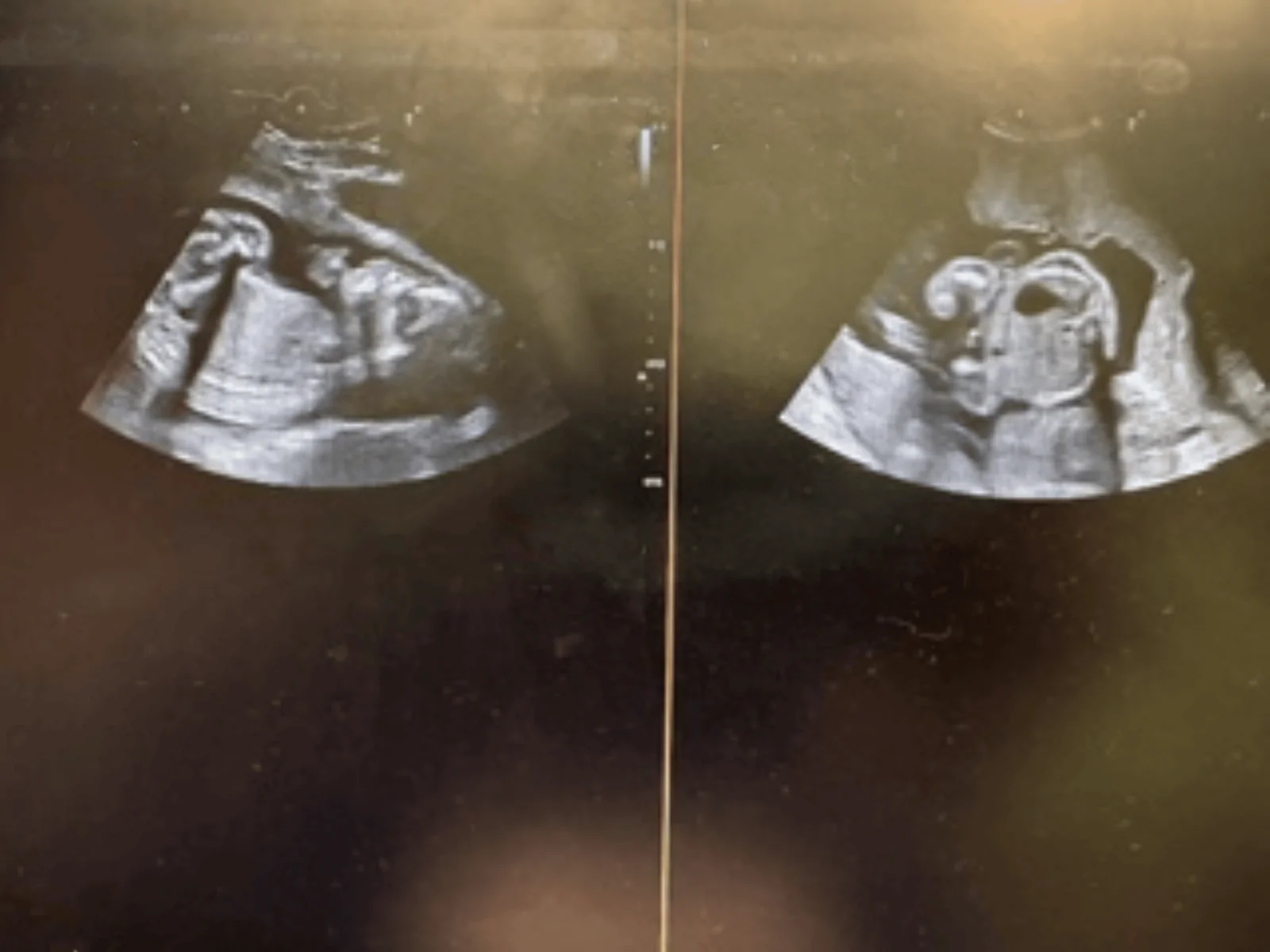
Twin pregnancy dichorionic diamniotic on ultrasound.

This is a case report of a 29-year-old woman who was in her second pregnancy and was diagnosed with a dichorionic diamniotic twin pregnancy (Figure 1).

**Figure 2:**
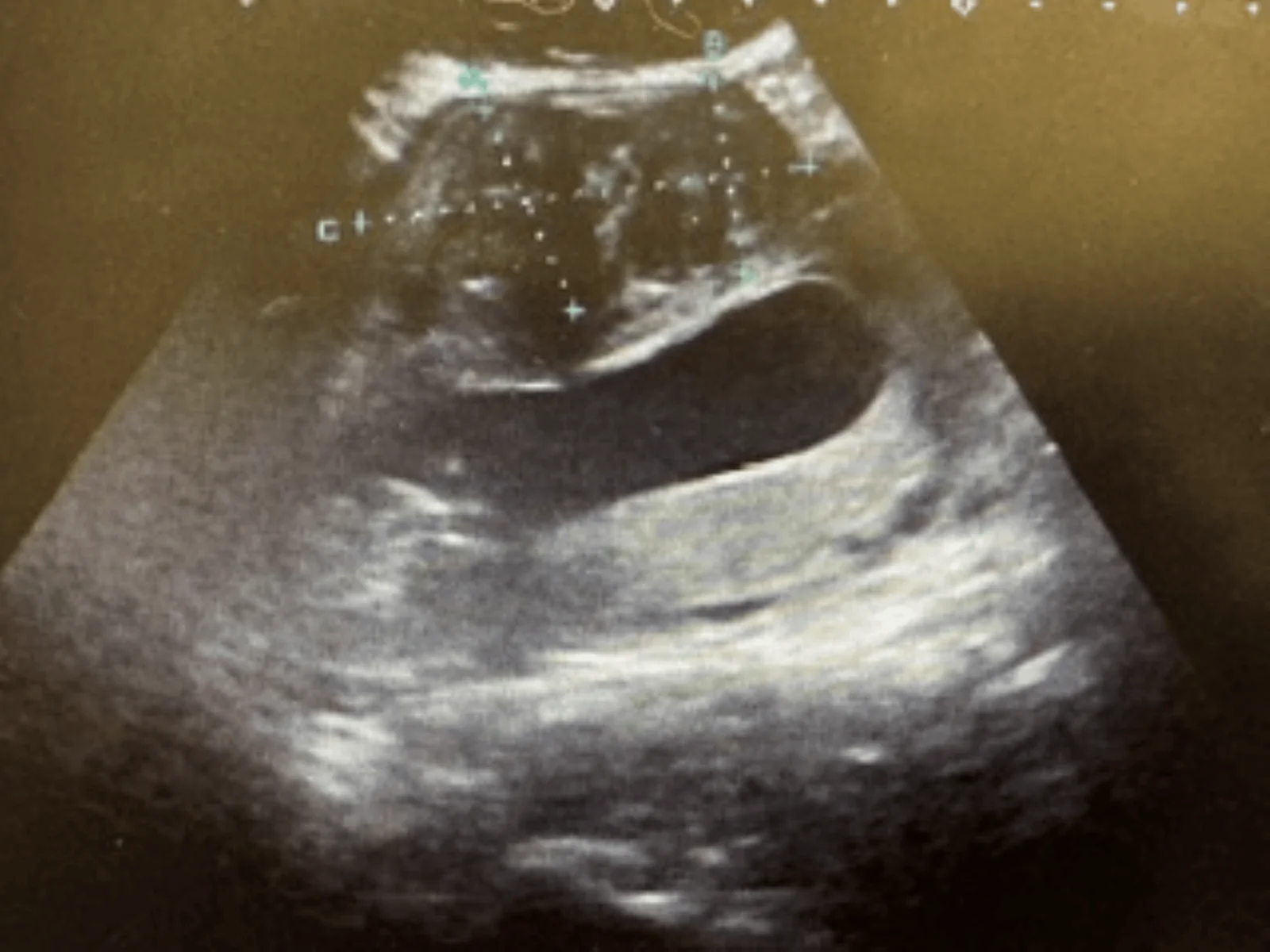
Anterior wall fibroid seen on ultrasound.

She had a Body Mass Index (BMI) of 27.68 and a venous thromboembolism (VTE) score of 1 [6]. Her ethnic origin was from Africa, and she had a previous normal birth at term in her own country. She gave a history of fibroids. The scan revealed two fibroids: one fundal, 4 x 5 cm, and another on the anterior wall, 3 x 4 cm (Figure 2). She had no symptoms of fibroids, with no evidence of pain or degeneration.

Routine antenatal blood investigations were normal except for positive hepatitis B serology. She was referred to a consultant gastroenterologist. Her liver profile was normal, and Hep C was negative. She was diagnosed with cervical incompetence at 17+3 weeks when a physical examination revealed a cervix dilated to 1-2 cm and a length of 1 cm with intact membranes and not bulging. She had an emergency McDonald suture applied under general anaesthesia with Mersilene tape in the form of a purse-string suture at the cervicovaginal junction in a clockwise direction, with all four ends passing through the cervix at approximately the 12, 3, 6, and 9 o'clock positions. It was then secured by tying it down anteriorly, resulting in adequate occlusion of the internal os without being overly tight. No complications were encountered, and she was prescribed the Cyclogest pessary, 400 mg, vaginally.

She had a background history of polycystic ovaries (PCOS) and a family history of diabetes. She was diagnosed with gestational diabetes during the investigations and was started on metformin, 1 g, orally once daily. The scan at 22+2 weeks revealed mild polyhydramnios for the second twin. She was found to be Group B Streptococcus (GBS) positive.

She was admitted at 27+3 weeks due to preterm premature rupture of membranes (PPROM), draining clear liquor, and being in established preterm labour. She was given an IV magnesium sulphate infusion for neuroprotection; IV antibiotics, including GBS cover; and steroids. She progressed from 3 to 5 cm dilatation.

She had an emergency category 1 caesarean section under spinal anaesthesia. Twin 1 was delivered as cephalic. Twin 2 had a transverse lie and was delivered as breech. There were two true knots in the umbilical cord of the second twin. Twin 1 weighed 970 grams and was extubated soon. Twin 2 weighed 900 grams, was ventilated, and then weaned off. Both twins were delivered in plastic bags. The estimated blood loss (EBL) was 300 mL. The cervical cerclage was removed vaginally.

Postoperatively, she developed breathlessness, and the clinical picture was suggestive of pulmonary oedema. The computed tomography pulmonary angiogram (CTPA) was negative for pulmonary embolism (PE), but her echocardiogram was suggestive of mild tricuspid, mitral, and aortic regurgitation. Overall cardiac function was optimal, with good biventricular function and an ejection fraction >55%. During the first month after the babies were born, they remained in the Special Care Baby Unit (SCBU), so she received regular home visits from the community midwife to continue postnatal care. The cardiac review took place at approximately three months postnatal; she was then discharged from cardiology follow-up with no further concerns. At 13 weeks postnatal, she was reviewed in primary care due to her diagnosis of gestational diabetes mellitus; no concerns were identified. The patient and her babies are doing well, as she told me during a recent telephone consultation.

## Discussion

Preterm delivery in twin pregnancies reflects a complex interplay of mechanical, inflammatory, and hormonal factors. Twin pregnancies now account for 3% of all pregnancies, an annual increase in occurrence brought on by assisted reproductive technologies. About 50% of twin pregnancies deliver at less than 37 weeks of gestation, and 14% of preterm births have been noted at less than 33 weeks of gestation. Furthermore, the rates of preterm births (PTBs) at less than 32 weeks of gestation are eight times greater than those of singletons. The burden on nations and families has increased significantly due to the rise in the prevalence of both short- and long-term newborn problems and mortality. Thus, concerns of utmost importance to obstetricians are preventing preterm birth and enhancing newborn outcomes [7].

More recent research has shown that cerclage may offer an advantage in some twin pregnancies due to structural changes in the cervix. The systematic review conducted by Liu et al. [8] demonstrated that perinatal outcomes improved and spontaneous preterm birth decreased among women who received cerclage for cervical shortening. Additionally, several observational studies reported that emergency cerclage in twins extended gestational age to a similar extent as in singleton deliveries [9]. Subsequent cohort studies have provided evidence that cerclage may be beneficial to neonatal outcomes in asymptomatic patients with a shortened cervix [10].

Uterine fibroids have been associated with an increased risk of adverse obstetric outcomes, including spontaneous preterm birth, malpresentation, and caesarean delivery [11]. While causality cannot be established from a single case, it is plausible that the combination of rescue cervical cerclage and vaginal progesterone supplementation contributed to prolonging the pregnancy by approximately 10 weeks, thereby improving neonatal outcomes [12,13].

Despite the promising results observed in this case, several challenges remain in the management of twin pregnancies with cervical insufficiency. The decision to proceed with cerclage must be individualised, taking into account gestational age, degree of cervical change, the presence of additional risk factors such as uterine fibroids, and the patient's preferences. Although the fibroids in this patient were relatively small, their coexistence with twin gestation and cervical insufficiency likely compounded the overall risk of spontaneous preterm birth [11-13].

However, several randomised trials and meta-analyses have found no significant benefit or even a possible increase in adverse outcomes when cerclage is used routinely in unselected twin pregnancies, particularly in those without clear evidence of cervical insufficiency. For instance, the meta-analysis by Berghella et al. and guidance from major obstetric societies caution against the universal use of cerclage in twin pregnancies due to a lack of proven efficacy and potential risks [5,14,15]. Thus, the current body of evidence is inconsistent due to differences in study designs and populations, as well as a paucity of robust randomised trials, so conclusions about efficacy should be drawn with caution.

Current guidelines from international societies do not universally recommend cerclage in unselected twin pregnancies, citing the lack of robust randomised controlled trial data and the potential for increased procedural risks. Rescue cerclage has shown promise in a few specific situations; however, it was used here due to cervical shortening/dilatation in the absence of labour or infection. Data from observational studies and meta-analyses indicate that rescue cerclage may be associated with a longer gestational age in appropriately selected patients; therefore, it may improve neonatal outcomes when performed in suitable candidates [5,8].

In addition, the multidisciplinary team approach (maternal-foetal medicine, neonatology, and anaesthesia) used in this patient's case provided optimal surveillance for potential complications and optimised maternal well-being and preparedness for the challenges of preterm birth. The multidisciplinary care model likely contributed to positive maternal and neonatal outcomes.

This case also demonstrates the value of using multiple modalities to manage high-risk pregnancy, including adjunctive therapy (vaginal progesterone) and administration of corticosteroids and magnesium sulphate at the time they are needed. Nevertheless, the interpretation of this case should acknowledge its limitations, including the inability to generalise outcomes from a single-patient experience and the inherent risk of bias. Large-scale, prospective studies are required to define patient selection criteria better and clarify the relative benefits and risks of rescue cerclage in twin gestations [14,15]. Until such data are available, clinical judgement, shared decision-making, and individualised care remain paramount.

## Conclusions

The case report mentions the role of a rescue cervical cerclage in twin pregnancy, but it is important to consider the holistic picture and give careful consideration. Following comprehensive counselling and shared decision-making, the patient elected to undergo rescue cerclage, which was associated with prolongation of pregnancy by approximately 10 weeks and survival of both neonates. However, the potential benefits of prolonging pregnancy must be carefully balanced against the procedural risks, including preterm premature rupture of membranes, infection, and pregnancy loss.

This case underscores the importance of individualised assessment, tailored care planning, and collaborative multidisciplinary team efforts, as well as the potential benefits of timely interventions in such clinical scenarios. Further high-quality prospective studies and randomised controlled trials are required to define better the indications, effectiveness, and safety of rescue cervical cerclage in twin pregnancies.
